# Perception of discrimination against immigrants compared to Chilean-born and its relationship with access to services and health outcomes

**DOI:** 10.11606/s1518-8787.2022056004125

**Published:** 2022-11-18

**Authors:** Marcela Oyarte, Báltica Cabieses, Manuel Espinoza, María Teresa Valenzuela, Iris Delgado

**Affiliations:** I Instituto de Salud Pública de Chile Departamento Agencia Nacional de Dispositivos Médicos, Innovación y Desarrollo Santiago Chile Instituto de Salud Pública de Chile. Departamento Agencia Nacional de Dispositivos Médicos, Innovación y Desarrollo. Santiago, Chile; II Universidad del Desarrollo Instituto de Ciencias e Innovación en Medicina Programa de Estudios Sociales en Salud Santiago Chile Universidad del Desarrollo. Instituto de Ciencias e Innovación en Medicina. Programa de Estudios Sociales en Salud. Santiago, Chile; III University of York Faculty of Health Department of Health Sciences York United Kingdom University of York. Faculty of Health. Department of Health Sciences. York, United Kingdom; IV Pontificia Universidad Católica de Chile Escuela de Medicina Departamento de Salud Pública Santiago Chile Pontificia Universidad Católica de Chile. Escuela de Medicina. Departamento de Salud Pública. Santiago, Chile; V Universidad de Los Andes Facultad de Medicina Departamento de Salud Pública y Epidemiología Santiago Chile Universidad de Los Andes. Facultad de Medicina. Departamento de Salud Pública y Epidemiología. Santiago, Chile; VI Universidad del Desarrollo Instituto de Ciencias e Innovación en Medicina Centro de Epidemiología y Políticas de Salud Santiago Chile Universidad del Desarrollo. Instituto de Ciencias e Innovación en Medicina. Centro de Epidemiología y Políticas de Salud. Santiago, Chile

**Keywords:** Emigrants and Immigrants, Racism, Social Perception, Health Services Accessibility, Contextual Effects of Health Disparities

## Abstract

**OBJECTIVES:**

Compare self-perceived discrimination between immigrants and locals in Chile and analyze the relationship between immigration and perceived discrimination and immigration, discrimination and health outcomes, adjusting for sociodemographic characteristics and social capital.

**METHODS:**

Cross-sectional study, using population-based survey (CASEN2017). We selected 2,409 immigrants (representative of N = 291,270) and 67,857 locals (representative of N = 5,438,036) over 18 years of age surveyed. We estimated logistic regression models, considering the complex sample, with discrimination, self-rated health, medical treatment, healthcare system membership, complementary health insurance, medical consultation and problems when consulting as dependent variables, immigration and discrimination as main exposure variables, and social capital and sociodemographic variables as covariates of the models.

**RESULTS:**

Immigrants were more likely to perceive discrimination in general compared to locals (OR = 2.31; 95%CI: 1.9-2.9). However, this does not occur for all specific reasons for discrimination; skin color and physical appearance were the most frequent causes of discrimination in immigrants. The interaction between immigration and discrimination was significantly related to worse self-rated health outcomes and treatment for pathologies, disfavoring discrimination against immigrants. In both locals and immigrants, discrimination was not associated with health care access outcomes, except for problems during consultation in locals (OR = 1.61; 95%CI 1.4-1.8).

**CONCLUSIONS:**

In Chile, experiences of discrimination are intertwined with other forms of rejection and social exclusion, so it is urgent to raise awareness among the population to prevent these discriminatory practices, especially in health care and daily use places. It is essential to address discrimination in order to have an impact on intermediate variables and health outcomes. The extension of the results to the entire immigrant population could be very useful to deepen the problem and improve the estimates made.

## INTRODUCTION

Discrimination is defined as different treatment of a person or group with common characteristics^1^; and it is a relevant structural factor in the development of inequalities in health, especially in minority social groups^2,3^. The perception of negative discrimination has effects on the general well-being and physical and mental health of the individual, which has been reported for various types of discrimination^3^. This can lead to poor access to quality medical care^2,4,7^ and hinder the scope of public health programs^3^. When differences between social groups are systematic, they produce social inequities in health that must be urgently addressed. In migrant populations this has been documented regardless of the development of the country, migration policy or characteristics of the health care system.

Regarding immigrants, studies based on ethnoracial discrimination have predominated, relating them to the presence of depressive symptoms, distress, low self-esteem^8,9^, higher prevalence of chronic diseases, poor general well-being, self-rated health, and adverse pregnancy and childbirth outcomes^9^. For example, in Moroccan immigrants settled in Spain, it was estimated that up to 40% of cases reported a deterioration in health that could be attributed to discrimination^10,11^. In Canada, afrodescendants and indigenous people have been more exposed to discriminatory experiences, exhibiting results suggesting that this is decisive in the presence of chronic diseases and their risk factors^12^. In some countries where legally guaranteed access to health care is available, barriers such as discriminatory practices, administrative requirements, language, fear of being reported and refusal of care hinder immigrants’ access to health care^13^.

Discrimination can damage health through multiple mechanisms^4,9,14^. In addition, acculturative stress^4,9,12^and a constant feeling of “otherness”^2,15^ can be triggers of physical-psychological discomfort in immigrants.

There are individual differences in the perception, coping and reception of discrimination, which can mitigate discrimination and its harmful effects^11^. Belonging to social or religious organizations and family, social and emotional support networks are frequently repeated as moderating factors in the discrimination and health-discrimination relationship^4,15^. Social and emotional supports show no positive effect on long-term disease management^15^ and protect against threats to physical-psychological health^2,15^, respectively. Variables such as age, sex, marital status, education, socioeconomic level, poverty, race and ethnicity are associated with both discrimination and health outcomes. In addition, time of stay, country of origin, and migratory status and distance are also important in the case of immigrants^16,17^.

In Latin America, studies on discrimination of the immigrant population and its relationship with health outcomes and access to health care are scarce, especially at the population level. Likewise, their understanding of potentially protective social processes, such as social capital, is limited.

Among the countries in the region, Chile experienced a sustained increase in international migration according to current estimates by the National Institute of Statistics, which reports 1,462,103 immigrants in the country by the end of 2020, reaching 8% of the total population^18^. Previous evidence highlighted the heterogeneity of this population, whose demographic and socioeconomic characteristics differ not only from the Chilean population, but also within migrant groups^19^. Although migrants are a heterogeneous group, seven nationalities accounted for approximately 79% of foreigners in 2020. First, Venezuelans (30.7%), followed by Peruvians (16.3%), Haitians (12.5%), Colombians (11.4%) and Bolivians (8.5%)^18^.

A recent study with the Peruvian and Colombian population residing in three cities in Chile (Arica, Antofagasta, Santiago) found a high presence of symptoms associated with mental health problems, mainly depression, anxiety, difficulties in social interaction and social adjustment problems, related to discrimination, high levels of acculturation stress and the use of acculturation strategies linked to assimilation and marginalization^20^. Although the country rectifies several international conventions and establishes non-discrimination^21,22^in its legislation, the National Institute of Human Rights recorded multiple complaints of discrimination against the immigrant population, in daily interactions or in collective actions by authorities and institutions^22^.

On the other hand, the Chilean Health System is segmented and fragmented with public and private participation. Subsystems with different financing and provision modalities coexist in it. International migrants showed lower access to this system in contrast to local migrants, an increasing gap, from 9% in 2013 to 18% in 2017^19^, although regular and irregular migrants guaranteed access to public health care by formal enrollment in a health center close to their place of living in the entire national territory, as stated in Decree 67 of 2016.

Consequently, this study aimed to describe inequality gaps in perceived discrimination between migrants and locals, in a crude form and adjusted for sociodemographic variables and social capital; as well as their possible association with access to the health system and overall health outcomes. The specific objectives of this study were: (i) compare self-perceived discrimination between immigrants and locals in Chile, (ii) explore the association of perceived discrimination with being an international migrant, in a crude manner and adjusted for sociodemographic, economic and social capital covariates, and (iii) analyze the relationship between access to health services and health outcomes (as response variables) and perceived discrimination in international migrants and the local population.

## METHODS

This is a cross-sectional analytical study, conducted from the National Socioeconomic Characterization Survey (Casen) of Chile in its 2017 version.

The Casen 2017 survey is a diagnostic, evaluation and targeting instrument aimed at understanding the socioeconomic conditions of households in Chile, especially in priority groups according to social policies. We collected data by means of structured interviews with qualified informants (heads of household or responsible adults), residents of private homes, excluding areas of difficult access. Casen 2017 uses a probabilistic, stratified and two-stage sampling, with national representativeness, which allows obtaining population sizes from expansion factors^23^.

The 2017 Casen survey collected information from 6,811 (representative of 777,407) international migrants, identified as those who self-reported being born in a country other than Chile, 207,603 (representative of 16,843,471) locals identified as those who self-reported being born in Chile, and 2,025 (representative of 186,536) who reported not knowing their country of birth (0.94% of total respondents).

The study included eligible informants who were international migrants (2,409 respondents, representative of 291,270 people) and local migrants (67,857 respondents, representative of 5,438,036 people), in accordance with the population that effectively responds to the perceived discrimination variable. According to the Casen survey, a suitable informant is defined as any head of household or, alternatively, a member of the household aged 18 years or older.

### Study Variables

Perceived discrimination:

1. Perceived discrimination (yes/no) based on: (a) socioeconomic status, (b) being a woman or a man, (c) marital status, (d) clothing, (e) skin color, (f) being a foreigner, (g) age, (h) sexual orientation or gender identity, (i) tattoos, piercings, plugs, (j) physical appearance, (k) beliefs or religion, (l) political ideology or opinion, (m) participation or not in unions or associations, (n) place where they live, (o) establishment where they studied, (p) belonging to an indigenous people, (q) health condition or disability, (r) other reasons. Corresponding to 18 independent variables.
2. Perceived discrimination on one or more reasons (any one/s of the above, binary = yes/no).

Access to the health system and its use:

1. General access, that is, enrollment in the social security health system (Yes: public, private or other system; No: not belonging, private care).
2. Complementary health coverage (Yes: any member of the family nucleus covered by Complementary Health Insurance (SSC – *Seguro de Salud Complementario*) for risk of illness or accident; No: no member covered by SSC).
3. Consultation in case of illness or accident (yes/no).
4. Consultation in case of illness or accident (multinomial = no consultation; consultation with at least one problem in care; consultation with no problems).

Health outcomes:

1. Medical treatment in the last year (yes/no).
2. Self-rated health (SAV – *Salud autoevaluada*), on a scale of 1 to 7, being 1 very bad and 7 very good.
3. Poor SAV (yes/no), based on SAV variable categorized as yes = 1,2 and no = 3,4,5,6,7.

Social capital:

1. Participation in organizations or organized groups (yes/no).
2. Social and/or family support networks (yes/no).

Sociodemographic variables: Sex, age, ethnicity, educational level, area, household income quintile, occupation. In immigrants: time of residence in Chile and country of origin.

### Statistical Analysis

We estimated the percentages of the discrimination against immigrant and local population, both in total and according to the reasons for discrimination. Likewise, we analyzed descriptively the variables of health outcomes, access and use of the health system, distinguishing between those who perceived discrimination and those who did not, using frequency measures with their respective confidence intervals. We analyzed the independence between variables using chi-square tests with Rao-Scott correction (Figure 1). On the other hand, the effect of migration on total and cause-specific perceived discrimination was analyzed using the OR, obtained from logistic regression models, crude and adjusted for sociodemographic variables and social capital.

**Figure:**
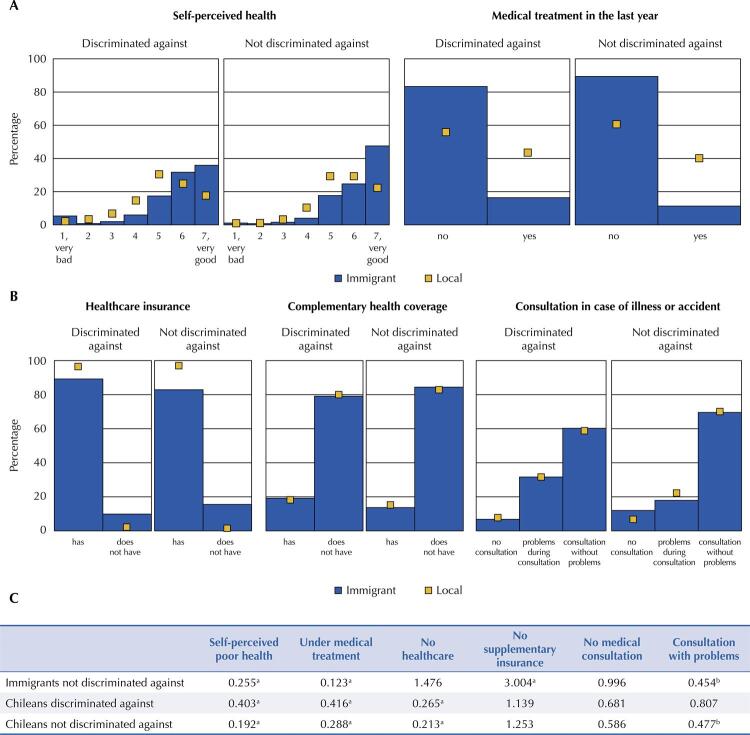
Distribution of health outcomes, in conjunction with OR of the intersection between discrimination and migrant status for (A) and access to health services (B), according to the perception of discrimination in immigrants and locals, along with logistic regression models (C). Chile, 2017.

Note: immigrants: p-value chi-square test with Rao-Scott correction (discrimination, self-rated health = 0.1307) (discrimination, medical treatment = 0.2097) (discrimination, healthcare insurance = 0.1769) (discrimination, complementary health coverage = 0.1805) (discrimination, medical consultation = 0.3383). Locales: p-value chi-square test with Rao-Scott correction (discrimination, self-rated health = 0.0000) (discrimination, medical treatment = 0.0000) (discrimination, healthcare insurance = 0.0154) (discrimination, complementary health coverage = 0.0000) (discrimination, medical consultation = 0.0000).

F test (Archer and Lemeshow): self-perceived poor health model: p-value = 0.171; medical treatment model: p-value < 0.001; healthcare insurance model: p-value = 0.224; complementary health insurance model: p-value = 0.283; no consultation model: p-value = 0.445; consultation with problems model: p-value = 0.098 (the model of consulting with or without problems is only used in the population that consults in the event of illness or accident).

^a^ p-value OR < 0.01.

^b^ p-value OR < 0.05, Wald test, logistic regression models.

Logistic regression models were fitted to analyze the effect of social capital and sociodemographic factors on perceived discrimination, using discrimination perception as the dependent variable and social support networks and participation in social organizations as independent variables, for immigrants and locals separately (models 1,3).


$$\text{ logit(discrimination })=\beta_{0}+\beta_{1}\text{ support networks }+\beta_{2}\text{ sacial participation }$$

Subsequently, we added to these models an adjustment for sociodemographic variables (va. SD) (models 2,4).


$$\text{ logit(discrimination) }=\beta_{0}+\beta_{1}\text{ support networks }+\beta_{2}\text{ social participation }+\beta \to \text{ va.SD }$$

Similarly, we fitted models of the form (models 5-10) to explore the effect of the interaction between immigration and perceived discrimination on the various health outcomes and access to health services:


$$\mathrm{logit}(\text{ SAV })=\beta_{0}+\beta_{1}\text{ migration }\#\text{ perceived discrimination }+\delta^{*}$$

$$\text{ logit(medical treatment })=\beta_{0}+\beta_{1}\text{ migration"perceived discrimination }+\delta^{*}$$

$$\text{ logit(healthcare insurance })=\beta_{0}+\beta_{1}\text{ migration perceived discrimination }+\delta^{*}$$

$$\text{ logit(medical consultation })=\beta_{0}+\beta_{1}\text{ migration }\#\text{ perceived discrimination }+\delta^{*}$$

$$\text{ logit(problems during consultation })=\beta_{0}+\beta_{1}\text{ migration }\neq \text{ perceived discrimination }+\delta^{*}$$

$$\delta^{*}=\beta_{2}\text{ support networks }+\beta_{3}\text{ social participation }+\beta \to \text{ va.SD }$$

Finally, we explored the relationship between discrimination and health outcomes and access to health in immigrants and locals separately, using form models:


$$\mathrm{logit}(SAV)=\beta_{0}+\beta_{1}\text{ perceived discrimination }+\delta^{*}$$

$$\mathrm{logit}(\text{ medical treatment })=\beta_{0}+\beta_{1}\text{ perceived discrimination }+\delta^{*}$$

$$\text{ logit(healthcare insurance })=\beta_{0}+\beta_{1}\text{ perceived discrimination }+\delta^{*}$$

$$\text{ logit }(\text{ medical consultation })=\beta_{0}+\beta_{\text{a perceived discrimination }}+\delta^{*}$$

$$\text{ logit }(\text{ problems during consultation })=\beta_{0}+\beta_{1}\text{ perceived discrimination }+\delta^{*}$$

$$\delta^{*}=\beta_{2}\text{ support networks }+\beta_{3}\text{ social participation }+\beta \to \text{ va.SD }$$

In immigrant we additionally adjust for country of origin and length of residence.

For all estimated models, the robustness of fit was examined by means of an adjusted F-test of residual means (Archer and Lemeshow).

All analyses were performed with Stata 14 software, with significance of 0.05, 95% confidence, considering the complex sample (strata, clusters, expansion factors), treating strata with a single cluster as a unit of certainty and Taylor linearization for variance estimation.

## RESULTS

### Migration and Perceived Discrimination

Of all eligible immigrant informants, 26.92% (95%CI: 21.5–33.2), equivalent to 78,413 people, reported being discriminated against or treated unfairly because they were foreigners. In immigrants, physical appearance and skin color were the most frequently reported reasons for discrimination, differing significantly from the local population. In crude models, immigrants were 8.23 (95%CI: 5.4–12.5) times more likely to be discriminated against because of their skin color compared to locals and 2.82 (95%CI: 1.4–5.8) times more likely to be discriminated against because of their physical appearance. After adjusting for social capital and sociodemographic variables, these magnitudes of association were 5.27 (95%CI: 3.4–8.1) and 1.71 (95%CI: 0.91–3.2), respectively. In contrast, immigrants were significantly less likely to report discrimination based on being male or female (OR = 0.53; 95%CI: 0.4–0.8), place where they live (OR = 0.43; 95%CI: 0.2–0.9), age (OR = 0.32; 95%CI: 0.2–0.5), religion (OR = 0.24; 95%CI: 0.1–0.5), sexuality or gender identity (OR = 0.34; 95%CI: 0.1–0.8), establishment where they studied (OR = 0.21; 95%CI: 0.1–0.5), and tattoos, piercings or plugs (OR = 0.16; 95%CI: 0.1–0.4), than locals. For all adjusted models, social support networks were statistically significant, generally being protective factors (Table 1).

**Table 1:** Perceived discrimination in immigrants and locals, according to reasons for discrimination, and measures of effect of being an immigrant or non-immigrant on perceived discrimination. Chile, 2017.

Discrimination because of:	International migrants% (IC95%)	Born in Chile% (IC95%)	OR model adjusted by		

			Migration	Migration + social capital	Migration + social capital + SD
Being a foreigner	26.92 (21.5–33.2)				
Physical appearance	6.56 (3.3–12.5)	2.42 (2.3–2.6)^f^	2.82^e^	2.67^c.e^	1.71^c^
Skin color	4.03 (2.9–5.6)	0.51 (0.4–0.6)^f^	8.23^e^	8.76^b.e^	5.27^a.e^
Ideology or political opinion	2.70 (0.7–10.0)	1.01 (0.9–1.1)	2.72	2.89^b^	1.4^a^
Socioeconomic level	2.51 (1.5–4.3)	3.15 (2.9–3.4)	0.79	0.78^c^	0.61^c^
Being male or female	2.26 (1.6–3.1)	2.22 (2.0–2.5)	1.02	1.07^c^	0.53^a.e^
Health condition or disability	2.23 (0.5–9.3)	2.00 (1.9–2.2)	1.12	1.04^c^	0.85^c^
Clothing	1.16 (0.6–2.3)	1.10 (1.0–1.3)	1.06	1.05^c^	0.61^c^
The place where they live	0.87 (0.5–1.6)	1.30 (1.1–1.5)	0.66	0.65^c^	0.43^c.d^
Age	0.77 (0.5–1.2)	1.91 (1.8–2.1)^f^	0.4^e^	0.41^b.e^	0.32^c.e^
Marital status	0.51 (0.2–1.2)	0.70 (0.6–0.8)	0.73	0.75	0.43^c^
Religious beliefs	0.36 (0.2–0.7)	0.95 (0.8–1.1)	0.38^e^	0.45^b.d^	0.24^a.e^
Sexuality or gender identity	0.30 (0.1–0.7)	0.39 (0.3–0.5)	0.77	0.76	0.34^c.d^
Establishment where they studied	0.22 (0.1–0.5)	0.50 (0.4–0.6)^f^	0.44	0.47	0.21^a.e^
Tattoos, piercings or plugs	0.20 (0.1 – 0.5)	0.47 (0.4–0.6)^f^	0.43	0.44	0.16^a.e^
Belonging to an indigenous people	0.14 (0.1–0.3)	0.40 (0.3–0.5)	0.35^e^	0.41^b.d^	0.53^a^
Participation or non-participation in unions or associations	0.11 (0.0–0.8)	0.16 (0.1–0.2)	0.66	0.77^b^	0.31^a^
Other reason (m)	1.48 (1.0–2.2)	2.50 (2.3–2.7)	0.59^e^	0.6^d^	0.41^c.e^
Discrimination (yes)	32.99 (28.1–38.3)	14.46 (13.9–15.0)^f^	2.91^e^	2.95^a.e^	2.31^a.e^

Note: discriminated against (yes): perceived discrimination for one or more of the reasons consulted; other reason (m): in international migrants among other reasons are reported: sexual harassment, abuse at work, attribution of responsibilities, family or ex-partner confrontation, convictions, personality traits, housing, profession or not having one, educational level, income, being pregnant or having children, having animals, being an orphan, lack of knowledge of the country’s idioms, lack of opportunities and discrimination or mistreatment in the workplace, hospitals, restaurants, airlines or obtaining a driver’s license.

Crude model: discrimination ~ migration; Social capital adjusted models: discrimination ~ migration + social participation + social support networks; Adjusted models: discrimination ~ migration + social participation + social support networks + sociodemographic variables.

OR: *odds ratio*; 95%IC: 95% confidence interval; SD: sociodemographic variables.

^a^ p-value OR_ social participation < 0.05 and p-value OR_support networks < 0.05.

^b^ p-value OR_ social participation < 0.05

^c^ p-value OR_ support networks < 0.05.

^d^ p-value OR_ immigrant < 0.05 (Wald test).

^e^ p = < 0.01 (immigrant) (Wald test).

^f^ F Rao-Scott test, p-value (migration, discrimination) < 0.05 considering the complex sample design.

Thirty-two point ninety-nine percent (95%CI: 28.08–38.31) of immigrants (n = 96,095) and 14.46% (95%CI: 13.91–15.02) of locals claimed to experience unfair treatment or discrimination outside their home. After adjusting for sociodemographic variables and social capital, immigrants were 2.3 (95%CI: 1.9–2.9) times more likely to be discriminated against than the local population (Table 1). In immigrants, age and area of residence were significant for perceived discrimination. In locals, all variables were significant, particularly, support networks showed a protective effect (OR = 0.77; 95%CI: 0.62–0.95) and those who participated in social organizations were 1.29 (95%CI: 1.20–1.39) times more likely to be discriminated against (Table 2).

**Table 2:** Measures of the effect of social capital and sociodemographic characteristics on perceived discrimination, in immigrants and locals. Chile, 2017.

	Model 1	Model 2	Model 3	Model 4
				
	OR (IC95%)	OR (IC95%)	OR (IC95%)	OR (IC95%)
Social participation				
Yes	0.733 (0.51–1.06)	0.809 (0.57–1.14)	1.204^a^ (1.12– 1.29)	1.286^b^ (1.20–1.38)
No	Ref.	Ref.	Ref.	Ref.
Support networks				
Yes	0.519 (0.16–1.74)	0.618 (0.29–1.29)	0.779^a^ (0.63–0.97)	0.599^b^ (0.51–0.69)
No	Ref.	Ref.	Ref.	Ref.
Sex				
Woman		1.283 (0.82–2.02)		1.387^b^ (1.29–1.45)
Man		Ref.		Ref.
Age		0.980 (0.96–0.99)		0.987^b^ (0.98–0.99)
Ethnicity				
Yes		0.873 (0.59–1.30)		0.828^b^ (0.75–0.92)
No		Ref.		Ref.
Educational level				
Elementary		1.579 (0.58–4.28)		0.695^b^ (0.60–0.81)
High School		1.609 (0.55–4.73)		0.644^b^ (0.55–0.76)
Higher		1.458 (0.59–3.60)		0.848 (0.72–0.99)^a^
Did not study		Ref.		Ref.
Area		0.675 (0.47–0.96)		0.650 (0.59–0.72)^b^
Rural				
Urban		Ref.		Ref.
Income quintile				
II		0.948 (0.61–1.48)		0.773 (0.71–0.84)^b^
III		1.251 (0.74–2.12)		0.743 (0.66–0.83)^b^
IV		0.866 (0.53–1.42)		0.706 (0.64–0.78)^b^
V (richer)		1.654 (0.72–3.79)		0.731 (0.65–0.82)^b^
I poorer		Ref.		Ref.
Occupation				
Unemployed		1.485 (0.67–3.28)		1.506 (1.28–1.78)^b^
Inactive		0.617 (0.36 – 1.07)		0.838 (0.76–0.92)^b^
Employed		Ref.		Ref.
Intercept	0.958 (0.29–3.11)		0.202 (0.16–0.25)	

Note: F test (Archer and Lemeshow): model 1: p-value = 0.971; model 2: p-value = 0.206; model 3: p-value = 1.000; model 4: p-value = 0.162. Models 2 and 4 were estimated without considering the constant.

Ethnicity: considers only the 9 indigenous peoples recognized by law in Chile; Occupation: employed (having a job), unemployed (not having a job but not interested in having one) and inactive (not interested in working).

OR: odds ratio; 95%CI: confidence interval; Ref: reference.

^a^ p-value OR < 0.05.

^b^ p-value OR < 0.01, Wald test.

### Migration, Discrimination, Access to Health and Health Outcomes

Descriptively, in the immigrant and local population, the discrimination against subgroup had a higher percentage of the population with poor health and in medical treatment compared to the non-discriminated against (Figure 1 A and B). After adjusting for social capital and sociodemographic variables, being a non-discriminated against immigrant decreased by 74.5% (OR = 0.255; 95%CI: 0.12–0.31) the chance of having poor SAV compared to immigrants who reported discrimination. Likewise, locals (discriminated against or not) have a significantly lower chance of having poor health compared to discrimination against immigrants. The same situation occurs for medical treatment, with an OR of 0.123 (95%CI: 0.08–0.19) in non-discrimination against immigrants, 0.416 (95%CI: 0.31–0.57) in discrimination against locals and 0.288 (95%CI: 0.21–0.32) in non- discrimination against locals (Figure 1 C).

In immigrants, having support networks reported 88.6% (95%CI: 0.06–0.23) and 67.2% (95%CI: 0.19–0.57) less chance of having poor SAV and being in treatment, respectively, compared to those without networks (Table 3).

**Table 3:** Logistic regression models of self-rated health (SAV: poor vs. fair or good) and medical treatment during the year prior to the survey (yes/no), adjusted for self-reported discrimination, social capital and sociodemographic characteristics, in immigrants and locals. Chile, 2017.

	Immigrants		Locals	
	
	SAV Model	Treatment model	SAV Model	Treatment model
			
	OR (IC95%)	OR (IC95%)	OR (IC95%)	OR (IC95%)
Discrimination				
Yes	1.276 (0.68–2.37)	1.992 (0.81–4.89)	1.743^a.b.c^ (1.52–2.00)	1.297 (1.21–1.39)^a.b.c^
No	Ref.	Ref.	Ref.	Ref.
Social participation				
Yes	0.859 (0.39–1.89)	1.613 (1.02–2.54)^b.c^	0.643 (0.57–0.73)^b^	1.137 (1.08–1.19)^b.c^
No (ref.)	Ref.	Ref.	Ref.	Ref.
Support networks				
Yes	0.114 (0.06–0.23)^b.c^	0.328 (0.19–0.57)^b.c^	0.156 (0.14–0.18)^b.c^	0.130 (0.09–0.12)^c^
No	Ref.	Ref.	Ref.	Ref.
Sex				
Woman	1.534 (0.78–2.99)	1.110 (0.65–1.89)	0.950 (0.84–1.07)	1.340 (1.26–1.42) ^c^
Man	Ref.	Ref.	Ref.	Ref.
Age	0.967 (0.94–0.99)^c^	1.047 (1.03–1.07)^c^	0.992 (0.99–0.99)^c^	1.042 (1.04–1.05)^c^
Ethnicity				
Yes	0.264 (0.10–0.71)^c^	0.112 (0.07–0.18)^c^	0.445 (0.40–0.50)^c^	0.652 (0.60–0.71)^c^
No	Ref.	Ref.	Ref.	Ref.
Educational level				
Did not study	0.253 (0.20–25.72)	0.100 (0.03–0.35)^c^	1.246 (0.92–1.69)	1.006 (0.86–1.18)
Elementary	2.058 (0.69–6.13)	0.418 (0.20–0.89)^c^	0.995 (0.84–1.18)	0.875 (0.81–0.95)^c^
High School	1.219 (0.63–2.36)	0.389 (0.24–0.64 ^c^	0.720 (0.62–0.83)^c^	0.742 (0.69–0.79)^c^
Higher	Ref.	Ref.	Ref.	Ref.
Area				
Rural	0.141 (0.31–0.65)^c^	0.863 (0.45–1.65)	0.690 (0.60–0.80)^c^	0.858 (0.80–0.92)^c^
Urban	Ref.	Ref.	Ref.	Ref.
Income quintile				
II	1.421 (0.50–4.07)	0.600 (0.32–1.41)	0.721 (0.62–0.84)^c^	0.801 (0.74–0.03)^c^
III	1.462 (0.58–3.68)	0.480 (0.28–0.82) ^c^	0.616 (0.52–0.73)^c^	0.820 (0.76–0.89)^c^
IV	0.829 (0.28–2.45)	0.342 (0.20–0.57)^c^	0.516 (0.44–0.61)^c^	0.773 (0.71–0.83)^c^
V richer	0.800 (0.16–3.91)	0.716 (0.33–1.57)	0.279 (0.22–0.36)^c^	0.665 (0.60–0.74)^c^
I poorest	Ref.	Ref.	Ref.	Ref.
Occupation				
Unemployed	6.662 (2.95–15.03)^c^	0.486 (0.15–1.53)	0.944 (0.67–1.34)	1.127 (0.99–1.28)
Inactive	4.034 (1.42–11.49)^c^	2.191 (1.26–3.80)^c^	2.290 (1.99–2.63)^c^	1.772 (1.67–1.88)^c^
Employed	Ref.	Ref.	Ref.	Ref.

Note: F test (Archer and Lemeshow): Immigrants: SAV models = 0.764; treatment model = 0.000; Locals: SAV models = 0.000; treatment model = 0.000. The models adjusted for sociodemographic variables were estimated without considering the constant.

Ethnicity: considers only the 9 native peoples recognized by law in Chile; Occupation: employed (having a job), unemployed (not having a job, but not interested in having one) and inactive (not interested in working).

SAV Model: self-rated health ~ discrimination + migration + social participation + social support networks + sociodemographic variables. Treatment Model: Being or having been in medical treatment ~ discrimination + migration + social participation + social support networks + sociodemographic variables.

OR: *odds ratio*; 95%CI: 95% confidence interval; SAV: self-rated health; Ref: reference.

^a^ p-value OR < 0.05 Wald test, model adjusted only for the discrimination variable.

^b^ p-value OR < 0.05 Wald test, model adjusted only for the variables discrimination, social participation and support networks.

^c^ p-value OR < 0.05 Wald test, model adjusted only for the variables discrimination, social participation, support networks and sociodemographic variables.

Regarding access to health care, the interaction between discrimination and migration was not significant in the lack of consultation in the event of illness or accident (p-value OR > 0.05). However, in the population that used medical consultations, immigrants (OR = 0.454; 95%CI: 0.21–0.99) and Chileans (OR = 0.477; 95%CI: 0.27–0.86) who were not discriminated against were significantly less likely to present problems during the consultation compared to discrimination against immigrants. Whether they perceive discrimination or not, immigrants were more likely to have no health insurance compared to those born in Chile. Specifically, discrimination against Chileans have 0.265 (95%CI: 0.17–0.41) times less chance of not having health insurance compared to discrimination against immigrants, in non- discrimination against Chileans this value was 0.212 (95%CI: 0.14–0.32) times (Figure 1C).

Regarding sociodemographic factors in relation to access to health care in immigrants, ethnicity was significant in the lack of health insurance (OR = 0.565) and possession of complementary insurance (OR = 5.801). Likewise, the area of residence was significant for lack of healthcare insurance (OR = 1.545), possession of complementary insurance (OR = 2.181), lack of consultation (OR = 0.181) and problems during the consultation process (OR = 0.233). Finally, belonging to the richest quintile compared to the poorest quintile was significant in the lack of complementary insurance (OR = 0.129) and no consultation (OR = 0.124) (Table 4).

**Table 4:** Logistic regression models, having social security health system (yes/no), any family member covered by complementary health insurance (yes/no), no consultation before illness or accident (yes/no), problems during consultation (yes/no), adjusted for self-reported discrimination, social capital and sociodemographic characteristics, in immigrants and locals. Chile, 2017.

			Immigrants			Locals		
		
	SPS model	SSC model	CS model	CCP model	SPS model	SSC model	CS model	CCP model
							
	OR (IC95%)	OR (IC95%)	OR (IC95%)	OR (IC95%)	OR (IC95%)	OR (IC95%)	OR (IC95%)	OR (IC95%)
Discrimination								
Yes	0.569 (0.30–1.09)	0.728 (0.46–1.15)	0.596 (0.24–1.46)	1.399 (0.60–3.28)	1.123 (0.90– 1.40)^a.b^	0.940 (0.84–1.05)^a.b^	1.098 (0.87–1.39)	1.607 (1.41–1.83)^a.b.c^
Social participation								
Yes	0.713 (0.42–1.20)	0.970 (0.63–1.49)^b^	1.535 (0.62–3.79)	1.601 (0.82–3.14)	0.798 (0.68–0.94)^b.c^	0.914 (0.85–0.98)	0.914 (0.76–1.09)	1.089 (0.98–1.20)^b^
Support networks								
Yes	0.833 (0.47–1.49)^b^	2.345 (1.37–4.03)^b.c^	2.528 (0.98–6.50)^b^	0.212 (0.07–0.69)^b.c^	0.221 (0.18–0.27)^c^	2.264 (1.99–2.58)^b^	0.284 (0.21–0.38)^b.c^	0.481 (0.39–0.59)^c^
Sex								
Woman	1.050 (0.46–2.42)	1.345 (0.87–2.07)	1.152 (0.50–2.66)	1.888 (0.87–4.08)	0.543 (0.46–0.63)^c^	1.329 (1.23–1.43)	0.742 (0.63–0.88)^c^	1.082 (0.98–1.20)
Age								
	0.988 (0.97–1.01)	0.993 (0.98–1.01)	0.995 (0.97–1.02)	1.007 (0.99–1.03)	0.977 (0.97–0.98)^c^	1.017 (1.01–1.02)	0.991 (0.99–1.00)^c^	0.999 (1.00–1.00)
Ethnicity								
Yes	0.565 (0.33–0.97)^c^	5.801 (3.53–9.53)^c^	0.386 (0.514–1.03)	0.576 (0.13–2.51)	0.641 (0.55–0.75)^c^	1.020 (0.91–1.14)	0.741 (0.60–0.91)^c^	0.704 (0.60–0.82)^c^
Educational level								
Did not studied	0.473 (0.10–2.15)	-	-	-	0.477 (0.26–0.88)^c^	8.022 (5.30–12.14)	0.576 (0.34–0.98)^c^	1.078 (0.79–1.46)
Elementary	0.891 (0.40–2.01)	3.881 (1.49–10.08)^c^	0.240 (0.05–1.11)	0.899 (0.30–2.68)	0.699 (0.55–0.89)^c^	4.494 (3.98–5.08)	0.883 (0.67–1.17)	1.214 (1.04–1.42)^c^
High	0.641 (0.34–1.21)	2.805 (1.51–5.20)^c^	0.363 (0.15–0.85)^c^	1.299 (0.58–2.91)	0.752 (0.61–0.93)^c^	2.357 (2.18–2.55)	0.895 (0.71–1.13)	1.124 (0.99–1.28)
Area								
Rural	1.545 (1.06 – 2.25)^c^	2.181 (0.95 – 5.01)^c^	0.181 (0.05 – 0.67)^c^	0.233 (0.12 – 0.46)^c^	1.043 (0.86–1.26)	1.350 (1.16–1.57)	1.138 (0.93–1.39)	0.879 (0.74–1.04)
Income quintile								
II	0.595 (0.33–1.08)	1.794 (0.79–4.07)	0.154 (0.03–0.84)^c^	1.149 (0.31–4.21)	0.737 (0.60–0.90)^c^	0.749 (0.66–0.84)	0.804 (0.65–0.99)^c^	0.898 (0.78–1.03)
III	0.693 (0.35–1.36)	1.197 (0.61–2.37)	0.804 (0.21–3.03)	0.766 (0.25–2.39)	0.768 (0.63–0.94)^c^	0.548 (0.49–0.62)	0.802 (0.65–1.00)^c^	0.822 (0.70–0.96)^c^
IV	1.087 (0.48–2.47)	0.570 (0.29–1.12)	0.425 (0.10–1.78)	0.609 (0.16–2.38)	0.690 (0.54–0.87)^c^	0.367 (0.33–0.41)	0.733 (0.55–0.98)^c^	0.736 (0.63–0.86)^c^
V (richer)	0.514 (0.26–1.03)	0.129 (0.08–0.22)^c^	0.124 (0.02–0.65)^c^	0.771 (0.19–3.19)	0.701 (0.51–0.96)^c^	0.173 (0.15–0.19)	0.577 (0.41–0.80)^c^	0.528 (0.43–0.64)^c^
Occupation								
Unemployed	1.939 (0.98–3.84)	1.530 (0.47–4.94)	-	3.322 (1.16–9.48)^c^	1.593 (1.17–2.16)^c^	2.180 (1.71–2.78)	0.689 (0.40–1.19)	0.987 (0.75–1.30)
Inactive	1.070 (0.50–2.28)	0.614 (0.31–1.22)	0.435 (0.13–1.46)	0.437 (0.13–1.52)	0.666 (0.53–0.84)^c^	1.375 (1.23–1.54)	0.971 (0.77–1.22)	1.146 (1.00–1.31)^c^

Note: SPS model: having a healthcare insurance ~ discrimination + migration + social participation + social support networks + sociodemographic variables. SSC model: any family member covered by complementary health insurance ~ discrimination + migration + social participation + social support networks + sociodemographic variables. CS model: no consultation in case of illness or accident ~ discrimination + migration + social participation + social support networks + sociodemographic variables. CCP model: problems during consultation ~ discrimination + migration + social participation + social support networks + sociodemographic variables.

Reference categories: discrimination, not discriminated against; social participation, does not participate; support networks, no networks; sex, male; ethnicity, does not belong to any; educational level, higher education; area, urban; quintile, I (poorest); occupation, employed.

OR: *odds ratio*; 95%CI: 95% confidence interval; SPS: no healthcare insurance; SSC: no household members with complementary health insurance; CS: no consultation in the event of illness or accident; CCP: consultation with problems (only in the consulting population).

^a^ p-value OR < 0.05; Wald test, discrimination model.

^b^ p-value OR < 0.05; Wald test, discrimination model and social capital.

^c^ p-value OR < 0.05; Wald test, model adjusted only for the variables discrimination, social participation, support networks and sociodemographic variables.

In contrast to immigrants, discrimination against locals was significant in presenting problems during the consultation process, to the disadvantage of those discriminated against [OR immigrants = 1.399 (95%CI: 0.60–3.28) versus OR locals = 1.604 (95%CI: 1.41–1.83)] (Table 4).

Finally, in immigrants, a longer time of residence in the country was a significant risk factor for being or having been under medical treatment (OR = 1.067) and a protective factor for not having health insurance, SSC (OR = 0.922) and lack of complementary health insurance (OR = 0.974). In the latter, country of origin also proved to be a significant factor (result not shown in table).

## DISCUSSION

This study aimed to describe inequality gaps in the perception of discrimination between immigrants and locals, from a social inequity approach to health. The results presented suggest that immigrants residing in Chile are more likely to perceive discrimination compared to locals, and that the interaction between immigration and discrimination may be related to worse self-rated health outcomes and medical treatment. According to the evidence, perceived discrimination could trigger a series of mechanisms that intervene in the risk of becoming ill, and can be distinguished between (i) direct, by provoking a response to stress and unhealthy coping with it; and (ii) indirect or structural, by limiting employment, income, general well-being, and access to and perceived treatment in health systems and services, among others^12^. Regarding the latter, in this study, although discrimination did not show a significant association with non-consultation, in some situations it was related to presenting problems during the consultation, which could have negative repercussions on seeking care in the future.

Some international studies showed similar results. For example, in Iraqi refugees and immigrants, the perception of discrimination contributed significantly to the prediction of depression and poor or fair self-rated health, increasing up to twice the risk in the discriminated against population^17^. In Ghanaian immigrants in Europe, higher levels of perceived discrimination were associated with a higher risk of cardiovascular disease^24^, and in immigrants of different origins using social and health services in Barcelona, approximately 50% of respondents reported feeling discriminated against in such services, worsening in irregular immigrants^25^.

In the current context, discrimination tends to occur as subtle or structural microaggressions, rather than explicit. Hence, perceived discrimination is used worldwide in similar studies, since its subjective nature makes it possible to capture events not defined as discriminatory according to local laws or particular social definitions^11,12^. Thus, in the immigrant population, social capital can play an ambiguous role in the perception of discrimination by protecting against and coping with discriminatory events, but it can facilitate the perception of subtle aggressions in accordance with the customs of the receiving country and isolation in closed groups.

In Chile, experiences of discrimination are intertwined with other forms of rejection, social exclusion, classism, racism and contempt for people living in poverty^21^. Among the study participants who perceived discrimination, approximately one third said they were discriminated against for multiple reasons. This shows the complexity of these multiple and dynamic social processes, as well as the importance of having coordinated and planned actions to protect the dignity, health and suppress any form of social disadvantage of immigrants. Considering the possible chronic effects of discrimination and the fact that the main reasons for discrimination among immigrants are perceived externally, it is urgent to raise awareness among the local population about its prevention and rejection, especially in health care.

The strengths of this study are the simultaneous analysis of social and health variables, and the use of a reliable and highly accepted population base in Chile, allowing acceptable overall accuracy. On the other hand, since this is an analysis of secondary data, the exclusion of groups of interest (residents of inaccessible areas and homeless people), the impossibility of analyzing other variables relevant to the problem, and limited sample sizes in the migrant population, which was not the focus of the sample selection, are limitations. Finally, we recognize the cross-sectional nature of the data, which prevents us from inferring causality.

The results of this study can support the development of public and health policies in Chile and in other countries that show similar results. There is evidence from Latin America and the Caribbean documenting the relationship between experiences of stigma, discrimination and racism and poorer health outcomes in migrants, refugees and moving people. These social phenomena are complex and dynamic, and relevant when it comes to delving into the social mechanisms of rejection and exclusion that trigger a series of subjective and objective health outcomes. Discrimination has its origin in the formation of social groups that perceive themselves differently from others, thus creating one or multiple “othernesses” of lower rank or caste, loaded with stereotypes and prejudices. Therefore, the greater perception of discrimination among migrants compared to locals in Chile illustrates the existence of patterns of social interaction that differentiate and subjugate migrants based on their status as foreigners, in addition, as suggested by the results of this study, to their skin color and physical appearance. In terms of public policy, this study reinforces the importance of addressing, containing and reversing processes of stigma and discrimination against migrants in Chile, which is also documented in other countries. These public policy recommendations can be organized around different levels of action, for example: (i) the political level, with the development of regulations and mechanisms to reject all forms of discrimination in public contexts, including the health system, (ii) community level, through awareness campaigns that promote respect for social and cultural diversity and the construction of inclusive and respectful communities, (iii) health system, through the development of continuous training mechanisms on migration, discrimination and health, the promotion of teams with intercultural competencies in health, and permanent campaigns for the explicit rejection and auditing of all forms of discrimination and violence in health care.

Regarding future research, there are three relevant aspects documented in the literature not sufficiently analyzed in this study: (i) migration variables critical to the experience of discrimination as regularity and refuge/asylum^13^, (ii) longitudinal life course approach and (iii) analysis of specific health problems. Likewise, an approach to social inequalities integrating the intersection with gender identity is relevant, as these are often derived from the dominant values of the host society and are exacerbated in women^4^. Therefore, this study covers multiple approaches, but can be further developed, especially in population-based surveys including an adequate representation of migrant groups.
