# Supplementary material for: Perception of discrimination against immigrants compared to Chilean-born and its relationship with access to services and health outcomes
Source: Rev Saude Publica. 2022 Nov 18;56:121. doi: 10.11606/s1518-8787.2022056004125 (PMC9749657; doi:10.11606/s1518-8787.2022056004125)
Supplement: Translation

# Translation: Percepción de discriminación en inmigrantes comparados a nacidos en Chile y su relación con acceso a servicios y resultados de salud

**Authors:** Marcela Oyarte, Báltica Cabieses, Manuel Espinoza, María Teresa Valenzuela, Iris Delgado

**Affiliations:** I Chile Instituto de Salud Pública de Chile. Departamento Agencia Nacional de Dispositivos Médicos, Innovación y Desarrollo. Santiago, Chile; II Chile Universidad del Desarrollo. Instituto de Ciencias e Innovación en Medicina. Programa de Estudios Sociales en Salud. Santiago, Chile; III United Kingdom University of York. Faculty of Health. Department of Health Sciences. York, United Kingdom; IV Chile Pontificia Universidad Católica de Chile. Escuela de Medicina. Departamento de Salud Pública. Santiago, Chile; V Chile Universidad de Los Andes. Facultad de Medicina. Departamento de Salud Pública y Epidemiología. Santiago, Chile; VI Chile Universidad del Desarrollo. Instituto de Ciencias e Innovación en Medicina. Centro de Epidemiología y Políticas de Salud. Santiago, Chile

**Keywords:** Emigrantes e Inmigrantes, Racismo, Percepción Social, Accesibilidad a los Servicios de Salud, Efectos Contextuales de las Desigualdades en Salud

## Abstract

**OBJETIVOS:**

Comparar la autopercepción de discriminación entre inmigrantes y locales en Chile y analizar la relación entre inmigración y discriminación percibida e inmigración, discriminación y resultados de salud, ajustando por características sociodemográficas y capital social.

**MÉTODOS:**

Estudio transversal, utilizando encuesta poblacional (CASEN2017). Se seleccionaron 2.409 inmigrantes (representativos de N = 291.270) y 67.857 locales (representativos de N = 5.438.036) mayores de 18 años encuestados. Se estimaron modelos de regresión logística, considerando la muestra compleja, con discriminación, salud autoevaluada, tratamiento médico, pertenencia al sistema de salud, seguros complementarios de salud, consulta médica y problemas al consultar como variables dependientes, inmigración y discriminación como variables de exposición principal, y capital social y variables sociodemográficas como covariables de los modelos.

**RESULTADOS:**

Inmigrantes tuvieron mayor posibilidad de percibir discriminación en general comparado con locales (OR = 2,31; IC95% 1,9–2,9). Sin embargo, esto no ocurre para todos los motivos específicos de discriminación; color de piel y apariencia física fueron las causas de discriminación más frecuentes en inmigrantes. La interacción entre inmigración y discriminación se relacionó significativamente con peores resultados de salud autoevaluada y tratamiento por patologías, en desfavor de los inmigrantes discriminados. Tanto en locales como en inmigrantes la discriminación no se asoció con resultados de acceso a atención en salud, excepto problemas durante la consulta en locales (OR = 1,61; IC95% 1,4–1,8).

**CONCLUSIONES:**

En Chile, las experiencias de discriminación se entrelazan con otras formas de rechazo y exclusión social, por lo cual es urgente concientizar a la población para prevenir estas prácticas discriminatorias, sobre todo en la atención en salud y lugares de uso cotidiano. Abordar la discriminación es indispensable para lograr impactar en variables intermedias y resultados de salud. La extensión de los resultados a toda la población inmigrante podría ser de amplia utilidad para profundizar la problemática y mejorar las estimaciones realizadas.

## INTRODUCCIÓN

La discriminación se define como el trato diferente hacia una persona o grupo con características en común^1^; y es un factor estructural relevante en el desarrollo de desigualdades en salud, especialmente en grupos sociales minoritarios^2,3^. La percepción de discriminación negativa tiene efectos sobre el bienestar general y salud física y mental del individuo, lo que ha sido reportado para diversos tipos de discriminación^3^. Esta puede llevar a un acceso deficiente a atención médica de calidad^2,4,7^ y obstaculizar el alcance de programas de salud pública^3^. Cuando las diferencias entre grupos sociales son sistemáticas, producen inequidades sociales en salud que deben ser atendidas con urgencia. En poblaciones migrantes esto ha sido documentado independientemente del desarrollo del país, política migratoria o características del sistema de salud.

En inmigrantes han predominado estudios basados en discriminación etnoracial, relacionándolos con presencia de síntomas depresivos, angustia, baja autoestima^8,9^, mayor prevalencia de enfermedades crónicas, escaso bienestar general, salud autoevaluada y resultados adversos de embarazo y parto^9^. Por ejemplo, en inmigrantes marroquíes asentados en España, se estimó que hasta un 40% de los casos se informó un deterioro en la salud que podía atribuirse a discriminación^10,11^. En Canadá, afrodescendientes y aborígenes han sido más expuestos a experiencias discriminatorias, exhibiendo resultados que sugieren que esta es determinante en la presencia de enfermedades crónicas y sus factores de riesgo^12^. En algunos países donde el acceso a atención medica está garantizado por ley, barreras como prácticas discriminatorias, requisitos administrativos, idioma, miedo a ser denunciados y negativas a ser atendidos obstaculizan el acceso a cuidados en salud en inmigrantes^13^.

La discriminación puede dañar la salud mediante múltiples mecanismos^4,9,14^. En inmigrantes adicionalmente, estrés aculturativo^4,9,12^ y sensación constante de “otredad”^2,15^ pueden ser factores desencadenantes de malestar físico-psicológico.

Existen diferencias individuales en la percepción, afrontamiento y recepción de la discriminación, que pueden amortiguar esta y sus efectos nocivos^11^. Particularmente, pertenecer a organizaciones sociales o religiosas y redes de apoyo familiar, social y emocional se repiten frecuentemente como los factores moderadores en la discriminación y relación salud-discriminación^4,15^. Los apoyos sociales y emocionales han mostrado afectar positivamente al tratamiento de enfermedades a largo plazo^15^ y proteger frente a amenazas a la salud físico-psicológica^2,15^, respectivamente. Las variables como edad, sexo, estado civil, educación, nivel socioeconómico, pobreza, raza y etnia se han asociado tanto a discriminación como a resultados de salud. En inmigrantes, adicionalmente, son importantes el tiempo de estadía, país de origen y situación y distancia migratoria^16,17^.

En Latinoamérica, estudios sobre discriminación en población inmigrante y su relación con resultados de salud y acceso a salud han sido escasos, más aún a niveles poblacionales. Igualmente, es limitada su comprensión con procesos sociales potencialmente protectores, como capital social.

Entre los países de la región, Chile ha experimentado un aumento sostenido de la migración internacional según las estimaciones actuales del Instituto Nacional de Estadística, que reporta 1.462.103 personas inmigrantes en el país a fines de 2020, alcanzando el 8% de la población total^18^. La evidencia previa ha resaltado la heterogeneidad de esta población, cuyas características demográficas y socioeconómicas no solo difieren de la población chilena, sino también dentro de los grupos migrantes^19^. Si bien los migrantes son un grupo heterogéneo, en 2020 siete nacionalidades constituían aproximadamente el 79% de los extranjeros. Primeramente, se encuentra los venezolanos (30,7%), seguido por peruanos (16,3%), haitianos (12,5%), colombianos (11,4%) y bolivianos (8,5%)^18^.

Un reciente estudio con la población peruana y colombiana residente en tres ciudades de Chile (Arica, Antofagasta, Santiago) reveló alta presencia de síntomas asociados a problemas de salud mental, fundamentalmente depresión, ansiedad, dificultades en interacción social y problemas de ajuste social; relacionándolos a discriminación, altos niveles de estrés por aculturación y utilización de estrategias de aculturación vinculadas a asimilación y marginación^20^. Si bien el país rectifica diversas convenciones internacionales y establece en su legislación la no discriminación^21,22^, el Instituto Nacional de Derechos Humanos ha registrado múltiples denuncias de discriminación hacia la población inmigrante, ya sea en interacciones cotidianas o como colectivo por parte de autoridades e instituciones^22^.

Por otra parte, el Sistema de Salud de Chile es segmentado y fragmentado con participación público y privada. En él coexisten subsistemas con distintas modalidades de financiamiento y provisión. Los migrantes internacionales han mostrado sostenidamente un menor acceso a este sistema en contraste con los locales, brecha que estaría aumentando, pasando del 9% en 2013 a un 18% en 2017^19^, aun cuando migrantes regulares y en situación irregular tienen garantía de acceso a la salud pública vía inscripción formal en un centro de salud cercano a su lugar de residencia en todo el territorio nacional según lo plantea el Decreto 67 del 2016.

En consiguiente, este estudio buscó describir brechas de desigualdad en percepción de discriminación entre migrantes y locales, en forma cruda y ajustada por variables sociodemográficas y capital social; así como su posible asociación con acceso al sistema de salud y resultados generales de salud. Los objetivos específicos de este estudio fueron: (i) comparar la autopercepción de discriminación entre inmigrantes y locales en Chile, (ii) explorar la asociación de la discriminación percibida con el ser migrante internacional, de manera cruda y ajustada por covariables sociodemográficas, económicas y capital social, y (iii) analizar la relación entre acceso a servicios de salud y resultados de salud (como variables respuestas) y discriminación percibida en migrantes internacionales y población local.

## MÉTODOS

Este es un estudio analítico de corte transversal, realizado a partir de la Encuesta de Caracterización Socioeconómica Nacional (Casen) de Chile en su versión 2017.

La encuesta Casen 2017 es un instrumento de diagnóstico, evaluación y focalización que busca conocer las condiciones socioeconómicas de los hogares en Chile, especialmente en grupos prioritarios según las políticas sociales. La recolección de datos se realizó mediante entrevistas estructuradas a informantes idóneos (jefes de hogar o adultos responsables), residentes de viviendas particulares, excluyendo áreas de difícil acceso. Casen 2017 utiliza un muestreo probabilístico, estratificado y bietápico, con representatividad a nivel nacional, que permite obtener tamaños poblacionales a partir de factores de expansión^23^.

La encuesta Casen 2017 recolectó la información de 6.811 (representativos de 777.407) migrantes internacionales, identificados como aquellos que autorreportan haber nacido en otro país distinto de Chile, 207.603 (representativos de 16.843.471) locales identificados como aquellos que autorreportaron haber nacido en Chile y 2.025 (representativos de 186.536) que reportaron no saber su país de nacimiento (0,94% de total de encuestados).

Se incluyeron en el estudio a informantes idóneos migrantes internacionales (2.409 encuestados representativos de 291.270 personas) y locales (67.857 encuestados, representativos de 5.438.036 personas), en concordancia con la población que responde efectivamente a la variable de discriminación percibida. Definiéndose, acorde a la encuesta Casen, como informante idóneo todo jefe(a) de hogar o, en su defecto, un integrante del hogar de 18 años o más.

### Variables de Estudio

Discriminación percibida:

1. I.Discriminación percibida (sí/no) por: (a) nivel socioeconómico, (b) ser mujer u hombre, (c) estado civil, (d) ropa, (e) color de piel, (f) ser extranjero, (g) edad, (h) orientación sexual o identidad de género, (i) tatuajes, piercing, perforaciones o expansiones, (j) apariencia física, (k) creencias o religión, (l) ideología u opinión política, (m) participar o no en sindicatos u organizaciones gremiales, (n) lugar donde vive, (o) establecimiento donde estudió, (p) pertenecer a un pueblo indígena, (q) condición de salud o discapacidad, (r) otros motivos. Correspondiente a 18 variables independientes.
2. II.Discriminación percibida por uno o más motivos (alguna/s de las anteriores,
3. binaria = sí/no).

Acceso y uso del sistema de salud:

1. I.Acceso general, esto es, inscripción al sistema previsional de salud (Sí: sistema público, privado u otro; No: no pertenecer, atención particular).
2. II.Cobertura de salud complementaria (Sí: algún miembro del núcleo familiar cubierto por Seguro de Salud Complementario (SSC) ante riesgo de enfermedad o accidente; No: ningún miembro cubierto por SSC).
3. III.Consulta ante enfermedad o accidente (sí/no).
4. IV.Consulta ante enfermedad o accidente (multinomial = no consulta; consulta con al menos un problema en la atención; consulta sin problemas).

Resultados de salud:

1. I.Tratamiento médico en el último año (sí/no).
2. II.Salud autoevaluada (SAV), en escala de 1 a 7, donde 1 es muy mal y 7 muy bien.
3. III.Mala SAV (sí/no), a partir de variable SAV categorizada en sí = 1,2 y no = 3,4,5,6,7.

Capital social:

1. I.Participación en organizaciones o grupos organizados (sí/no).
2. II.Redes de apoyo social y/o familiar (sí/no).

Variables sociodemográficas: Sexo, edad, pertenencia étnica, nivel educacional, zona, quintil de ingreso del hogar, ocupación. En inmigrantes: tiempo de residencia en Chile y país de origen.

### Análisis Estadístico

Se estimaron los porcentajes de población inmigrante y local discriminada total y según motivos de discriminación. Asimismo, se analizaron descriptivamente las variables de resultados de salud, acceso y uso del sistema de salud distinguiendo entre quienes percibieron discriminación y quienes no, utilizando medidas de frecuencia con sus respectivos intervalos de confianza. La independencia entre variables se analizó mediante pruebas chi cuadrado con corrección Rao-Scott (Figura 1). Por otra parte, el efecto de la migración sobre la discriminación percibida total y por causas específicas fue analizado mediante el OR, obtenido a partir de modelos de regresión logística, crudos y ajustados por variables sociodemográficas y capital social.

**Figura 1:** Distribución de los resultados de salud, en conjunto con los OR de la intersección entre discriminación y condición migrante para (A) y acceso a servicios de salud (B), según la percepción de discriminación en inmigrantes y locales, junto con los modelos de regresión logística (C). Chile, 2017.

Nota: inmigrantes: p-valor prueba de chi cuadrado con corrección de Rao-Scott (discriminación, salud autoevaluada = 0,1307) (discriminación, tratamiento médico = 0,2097) (discriminación, previsión de salud = 0,1769) (discriminación, cobertura complementaria de salud = 0,1805) (discriminación, consulta médica = 0,3383). Locales: p-valor prueba de chi cuadrado con corrección de Rao-Scott (discriminación, salud autoevaluada = 0,0000) (discriminación, tratamiento médico = 0,0000) (discriminación, previsión de salud = 0,0154) (discriminación, cobertura complementaria de salud = 0,0000) (discriminación, consulta médica = 0,0000).

F (Archer y Lemeshow): modelo mala salud autopercibida: p-valor = 0,171; modelo tratamiento médico: p-valor < 0,001; modelo previsión de salud: p-valor = 0,224; modelo seguro de salud complementario: p-valor = 0,283; modelo no consulta: p-valor = 0,445; modelo consulta con problemas: p-valor = 0,098 (el modelo de consultar con o sin problemas se realiza solo en población que consulta ante enfermedad o accidente).

^a^ p-valor OR < 0,01.

^b^ p-valor OR < 0,05, prueba de Wald, modelos de regresión logística.

Para analizar el efecto del capital social y los factores sociodemográficos sobre la discriminación percibida, en primera instancia, se ajustaron modelos de regresión logística utilizando como variable dependiente la percepción de discriminación y como independientes las redes de apoyo social y participación en organizaciones sociales, para inmigrantes y locales por separado (modelos 1,3).


$$\text{ logit }(\text{ discriminación })=\beta_{0}+\beta_{1}\text{ redes de apoyo }+\beta_{2}\text{ participación social }$$

Posteriormente, se adicionó a tales modelos un ajuste por variables sociodemográficas (va. SD) (modelos 2,4).


$$\text{ logit }(\text{ discriminación })=\beta_{0}+\beta_{1}\text{ redes de apoyo }+\beta_{2}\text{ participación social }$$

En la misma línea, para explorar el efecto de la interacción entre inmigración y discriminación percibida sobre los diversos resultados de salud y acceso a servicios de salud, se ajustaron modelos de la forma (modelos 5-10):


$$\mathrm{logit}(SAV)=\beta_{0}+\beta_{1}\text{ migración }\#\text{ discriminación percibida }+\delta^{*}$$

$$\text{ logit(tratamiento médico })=\beta_{0}+\beta_{1}\text{ migración }\#\text{ discriminación percibida }+\delta^{*}$$

$$\text{ logit (previsión de salud })=\beta_{0}+\beta_{1}\text{ migración \textbackslash{}\#discriminación percibida }+\delta^{*}$$

$$\text{ logit }(\text{ consulta médica })=\beta_{0}+\beta_{1}\text{ migración\textbackslash{}\#discriminación percibida }+\delta^{*}$$

$$\text{ logit (problemas durante la consulta) }=\beta_{0}+\beta_{1}\text{ migración }\#\text{ discriminación percibida }+\delta$$

$$\text{ Siendo }\delta^{*}=\beta_{2}\text{ redes de apoyo }+\beta_{3}\text{ participación social }+\beta \to \text{ va.SD }$$

Finalmente, se exploró la relación entre discriminación y resultados de salud y acceso a salud en inmigrantes y locales por separado, mediante modelos de la forma:


$$\mathrm{logit}(SAV)=\beta_{0}+\beta_{1}\text{ discriminación percibida }+\delta^{*}$$

$$\text{ logit(tratamiento médico })=\beta_{0}+\beta_{1}\text{ discriminación percibida }+\delta^{*}$$

$$\text{ logit(previsión de salud })=\beta_{0}+\beta_{1}\text{ discriminación percibida }+\delta^{*}$$

$$\text{ logit }(\text{ consulta médica })=\beta_{0}+\beta_{1}\text{ discriminación percibida }+\delta^{*}$$

$$\text{ logit (problemas durante la consulta })=\beta_{0}+\beta_{1}\text{ discriminación percibida }+\delta^{*}$$

$$\text{ Siendo }\delta^{*}=\beta_{2}\text{ redes de apoyo }+\beta_{3}\text{ participación social }+\beta \to \text{ va.SD }$$

En inmigrante se ajustó adicionalmente por país de origen y tiempo de residencia.

Para todos los modelos estimados, la bondad de ajuste fue examinada mediante *test* F ajustado de medias residuales (Archer y Lemeshow).

Los análisis se realizaron con el software Stata 14, con significancia de 0,05, confianza del 95%, considerando la muestra compleja (estratos, conglomerados, factores de expansión), tratando estratos con un único conglomerado como una unidad de certeza y linealización de Taylor para estimación de la varianza.

## RESULTADOS

### Migración y Discriminación Percibida

Del total de informantes idóneos inmigrantes, el 26,92% (IC95% 21,5–33,2), equivalente a 78.413 personas, indicó haber sido discriminado o tratado injustamente por ser extranjero. En inmigrantes, apariencia física y color de piel fueron los motivos de discriminación reportados con mayor frecuencia, difiriendo significativamente de la población local. En modelos crudos, los inmigrantes tuvieron 8,23 (IC95% 5,4–12,5) veces más chance de ser discriminados por su color de piel comparado con locales y 2,82 (IC95% 1,4–5,8) veces más chance de ser discriminados por su apariencia física. Tras ajustar por capital social y variables sociodemográficas, estas magnitudes de asociación fueron 5,27 (IC95% 3,4–8,1) y 1,71 (IC95% 0,91–3,2), respectivamente. Contrariamente, los inmigrantes tuvieron significativamente menos chance de reportar discriminación por ser hombre o mujer (OR = 0,53; IC95% 0,4–0,8), lugar donde vive (OR = 0,43; IC95% 0,2–0,9), edad (OR = 0,32; IC95% 0,2–0,5), religión (OR = 0,24; IC95% 0,1–0,5), sexualidad o identidad de género (OR = 0,34; IC95% 0,1–0,8), establecimiento donde estudió (OR = 0,21; IC95% 0,1–0,5) y tatuajes o perforaciones (OR = 0,16; IC95% 0,1–0,4), que los locales. Para todos los modelos ajustados, las redes de apoyo social fueron estadísticamente significativas, siendo generalmente factores protectores (Tabla 1).

**Tabla 1:** Discriminación percibida en inmigrantes y locales, según motivos de discriminación, y medidas de efecto del ser o no inmigrante sobre la discriminación percibida. Chile, 2017.

Discriminado por:	Migrantes internacionales % (IC95%)	Nacidos en Chile % (IC95%)	OR modelo ajustado por		

			Migración	Migración + capital social	Migración + capital social + SD
Ser extranjero	26,92 (21,5–33,2)				
Apariencia física	6,56 (3,3–12,5)	2,42 (2,3–2,6)^f^	2,82^e^	2,67^c,e^	1,71^c^
Color de piel	4,03 (2,9–5,6)	0,51 (0,4–0,6)^f^	8,23^e^	8,76^b,e^	5,27^a,e^
Ideología u opinión política	2,70 (0,7–10,0)	1,01 (0,9–1,1)	2,72	2,89^b^	1,4^a^
Nivel socioeconómico	2,51 (1,5–4,3)	3,15 (2,9–3,4)	0,79	0,78^c^	0,61^c^
Ser hombre o mujer	2,26 (1,6–3,1)	2,22 (2,0–2,5)	1,02	1,07^c^	0,53^a,e^
Condición de salud o discapacidad	2,23 (0,5–9,3)	2,00 (1,9–2,2)	1,12	1,04^c^	0,85^c^
Ropa	1,16 (0,6–2,3)	1,10 (1,0–1,3)	1,06	1,05^c^	0,61^c^
El lugar donde vive	0,87 (0,5–1,6)	1,30 (1,1–1,5)	0,66	0,65^c^	0,43^c,d^
Edad	0,77 (0,5–1,2)	1,91 (1,8–2,1)^f^	0,4^e^	0,41^b,e^	0,32^c,e^
Estado civil	0,51 (0,2–1,2)	0,70 (0,6–0,8)	0,73	0,75	0,43^c^
Creencias religiosas	0,36 (0,2–0,7)	0,95 (0,8–1,1)	0,38^e^	0,45^b,d^	0,24^a,e^
Sexualidad o identidad de género	0,30 (0,1–0,7)	0,39 (0,3–0,5)	0,77	0,76	0,34^c,d^
Establecimiento donde estudió	0,22 (0,1–0,5)	0,50 (0,4–0,6)^f^	0,44	0,47	0,21^a,e^
Tatuaje, perforaciones o expansiones	0,20 (0,1 – 0,5)	0,47 (0,4–0,6)^f^	0,43	0,44	0,16^a,e^
Pertenecer a un pueblo indígena	0,14 (0,1–0,3)	0,40 (0,3–0,5)	0,35^e^	0,41^b,d^	0,53^a^
Participar o no en sindicatos o gremios	0,11 (0,0–0,8)	0,16 (0,1–0,2)	0,66	0,77^b^	0,31^a^
Otro motivo (m)	1,48 (1,0–2,2)	2,50 (2,3–2,7)	0,59^e^	0,6^d^	0,41^c,e^
Discriminado (sí)	32,99 (28,1–38,3)	14,46 (13,9–15,0)^f^	2,91^e^	2,95^a,e^	2,31^a,e^

Nota: discriminado (sí): percibió discriminación por uno o más de los motivos consultados; otro motivo (m): en migrantes internacionales entre otros motivos se reportan: acoso sexual, abuso laboral, atribución de responsabilidades, confrontación familiar o exparejas, convicciones, cualidades propias de personalidad, vivienda, profesión o no tener una, nivel educacional, aporte de dinero, estar embarazada o tener hijos, tener animales, ser huérfano, desconocimiento de modismos del país, falta de oportunidades y discriminación o maltrato en el lugar de trabajo, hospitales, restaurantes, aerolíneas u obtención de licencia de conducir.

Modelo crudo: discriminación ~ migración; Modelos ajustado por capital social: discriminación ~ migración + participación social + redes de apoyo social; Modelos ajustado: discriminación ~ migración + participación social + redes de apoyo social + variables sociodemográficas.

OR: *odds ratio*; IC95%: intervalo de confianza del 95%; SD: variables sociodemográficas.

^a^ p-valor OR_participación social < 0,05 y p-valor OR_redes de apoyo < 0,05.

^b^ p-valor OR_participación social < 0,05

^c^ p-valor OR_redes de apoyo < 0,05.

^d^ p-valor OR_inmigrante < 0,05 (prueba de Wald).

^e^ p = < 0,01 (inmigrante) (prueba de Wald).

^f^ F *Test* Rao-Scott, p-valor (migración, discriminación) < 0,05 considerando el diseño complejo de la muestra.

El 32,99% (IC95% 28,08–38,31) de los inmigrantes (n = 96.095) y 14,46% (IC95% 13,91–15,02) de los locales afirmaron haber sido tratados injustamente o discriminados fuera del hogar. Tras ajustes por variables sociodemográficas y capital social, los inmigrantes tuvieron 2,3 (IC95% 1,9–2,9) veces más chance de haber sido discriminados que la población local (Tabla 1). En inmigrantes, edad y zona de residencia resultaron ser significativos para la discriminación percibida. En locales, todas las variables lo fueron, particularmente, las redes de apoyo mostraron un efecto protector (OR = 0,77; IC95% 0,62–0,95) y quienes participaban en organizaciones sociales tuvieron 1,29 (IC95% 1,20–1,39) veces más chance de ser discriminados (Tabla 2).

**Tabla 2:** Medidas de efecto del capital social y características sociodemográficas sobre la discriminación percibida, en inmigrantes y locales. Chile, 2017.

	Modelo 1	Modelo 2	Modelo 3	Modelo 4
			
	OR (IC95%)	OR (IC95%)	OR (IC95%)	OR (IC95%)
Participación social				
Si	0,733 (0,51–1,06)	0,809 (0,57–1,14)	1,204^a^ (1,12– 1,29)	1,286^b^ (1,20–1,38)
No	Ref.	Ref.	Ref.	Ref.
Redes de apoyo				
Si	0,519 (0,16–1.74)	0,618 (0,29–1,29)	0,779^a^ (0,63–0,97)	0,599^b^ (0,51–0,69)
No	Ref.	Ref.	Ref.	Ref.
Sexo				
Mujer		1,283 (0,82–2,02)		1,387^b^ (1,29–1,45)
Hombre		Ref.		Ref.
Edad		0,980 (0,96–0,99)		0,987^b^ (0,98–0,99)
Etnia				
Si		0,873 (0,59–1,30)		0,828^b^ (0,75–0,92)
No		Ref.		Ref.
Nivel educacional				
Básica		1,579 (0,58–4,28)		0,695^b^ (0,60–0,81)
Media		1,609 (0,55–4,73)		0,644^b^ (0,55–0,76)
Superior		1,458 (0,59–3,60)		0,848 (0,72–0,99)^a^
Sin educación		Ref.		Ref.
Zona		0,675 (0,47–0,96)		0,650 (0,59–0,72)^b^
Rural				
Urbano		Ref.		Ref.
Quintil de ingreso				
II		0,948 (0,61–1,48)		0,773 (0,71–0,84)^b^
III		1,251 (0,74–2,12)		0,743 (0,66–0,83)^b^
IV		0,866 (0,53–1,42)		0,706 (0,64–0,78)^b^
V (más rico)		1,654 (0,72–3,79)		0,731 (0,65–0,82)^b^
I, más pobre		Ref.		Ref.
Ocupación				
Desocupados		1,485 (0,67–3,28)		1,506 (1,28–1,78)^b^
Inactivos		0,617 (0,36 – 1,07)		0,838 (0,76–0,92)^b^
Ocupados		Ref.		Ref.
Intercepto	0,958 (0,29–3,11)		0,202 (0,16–0,25)	

Nota: F (Archer y Lemeshow): modelo 1: p-valor = 0,971; modelo 2: p-valor = 0,206; modelo 3: p-valor = 1,000; modelo 4: p-valor = 0,162. Los modelos 2 y 4 se estimaron sin considerar la constante.

Etnia: considera solo a los 9 pueblos originarios reconocidos por ley en Chile; Ocupación: ocupado (tener trabajo), desocupados (no tener trabajo pero no tiene interés en tenerlo) e inactivos (no tiene interés en trabajar).

OR: ; IC95%: intervalo de confianza del 95%; Ref.: referencia.

^a^ p-valor OR < 0,05.

^b^ p-valor OR < 0,01, prueba de Wald.

### Migración, Discriminación, Acceso a Salud y Resultados de Salud

Descriptivamente, en población inmigrante y local, el subgrupo de discriminados tuvo mayor porcentaje de población con mala salud y en tratamiento médico comparado con los no discriminados (Figura 1 A y B). Tras ajustar por capital social y variables sociodemográficas, el ser inmigrante no discriminado disminuyó en un 74,5% (OR = 0,255; IC95% 0,12–0,31) el chance de tener mala SAV en comparación con inmigrantes que reportaron discriminación. Asimismo, locales (discriminados o no) disponen significativamente de menor chance de tener mala salud en comparación con inmigrantes discriminados. Misma situación ocurre con el haber estado en tratamiento médico, con un OR de 0,123 (IC95% 0,08–0,19) en inmigrantes no discriminados, 0,416 (IC95% 0,31–0,57) en locales discriminados y 0,288 (IC95% 0,21–0,32) en locales no discriminados (Figura 1 C).

En inmigrantes, tener redes de apoyo reportaron un 88,6% (IC95%_OR_: 0,06–0,23) y 67,2% (IC95%_OR_: 0,19–0,57) menos chance de tener mala SAV y estar en tratamiento, respectivamente, comparados con aquellos sin redes (Tabla 3).

**Tabla 3:** Modelos de regresión logística de salud autoevaluada (SAV: mala . regular o buena) y tratamiento médico durante el año previo a la encuesta (sí/no), ajustado por discriminación auto-informada, capital social y características sociodemográficas, en inmigrantes y locales. Chile, 2017.

	Inmigrantes		Locales	
	
	Modelo SAV	Modelo tratamiento	Modelo SAV	Modelo tratamiento
			
	OR (IC95%)	OR (IC95%)	OR (IC95%)	OR (IC95%)
Discriminación				
Sí	1,276 (0,68–2,37)	1,992 (0,81–4,89)	1,743^a,b,c^ (1,52–2,00)	1,297 (1,21–1,39)^a,b,c^
No	Ref.	Ref.	Ref.	Ref.
Participación social				
Sí	0,859 (0,39–1,89)	1,613 (1,02–2,54)^b,c^	0,643 (0,57–0,73)^b^	1,137 (1,08–1,19)^b,c^
No (ref.)	Ref.	Ref.	Ref.	Ref.
Redes de apoyo				
Sí	0,114 (0,06–0,23)^b,c^	0,328 (0,19–0,57)^b,c^	0,156 (0,14–0,18)^b,c^	0,130 (0,09–0,12)^c^
No	Ref.	Ref.	Ref.	Ref.
Sexo				
Mujer	1,534 (0,78–2,99)	1,110 (0,65–1,89)	0,950 (0,84–1,07)	1,340 (1,26–1,42) ^c^
Hombre	Ref.	Ref.	Ref.	Ref.
Edad	0,967 (0,94–0,99)^c^	1,047 (1,03–1,07)^c^	0,992 (0,99–0,99)^c^	1,042 (1,04–1,05)^c^
Etnia				
Sí	0,264 (0,10–0,71)^c^	0,112 (0,07–0,18)^c^	0,445 (0,40–0,50)^c^	0,652 (0,60–0,71)^c^
No	Ref.	Ref.	Ref.	Ref.
Nivel educacional				
Sin educación	0,253 (0,20–25,72)	0,100 (0,03–0,35)^c^	1,246 (0,92–1,69)	1,006 (0,86–1,18)
Básica	2,058 (0,69–6,13)	0,418 (0,20–0,89)^c^	0,995 (0,84–1,18)	0,875 (0,81–0,95)^c^
Media	1,219 (0,63–2,36)	0,389 (0,24–0,64 ^c^	0,720 (0,62–0,83)^c^	0,742 (0,69–0,79)^c^
Superior	Ref.	Ref.	Ref.	Ref.
Zona				
Rural	0,141 (0,31–0,65)^c^	0,863 (0,45–1,65)	0,690 (0,60–0,80)^c^	0,858 (0,80–0,92)^c^
Urbano	Ref.	Ref.	Ref.	Ref.
Quintil de ingreso				
II	1,421 (0,50–4,07)	0,600 (0,32–1,41)	0,721 (0,62–0,84)^c^	0,801 (0,74–0,03)^c^
III	1,462 (0,58–3,68)	0,480 (0,28–0,82) ^c^	0,616 (0,52–0,73)^c^	0,820 (0,76–0,89)^c^
IV	0,829 (0,28–2,45)	0,342 (0,20–0,57)^c^	0,516 (0,44–0,61)^c^	0,773 (0,71–0,83)^c^
V (más rico)	0,800 (0,16–3,91)	0,716 (0,33–1,57)	0,279 (0,22–0,36)^c^	0,665 (0,60–0,74)^c^
I, más pobre	Ref.	Ref.	Ref.	Ref.
Ocupación				
Desocupados	6,662 (2,95–15,03)^c^	0,486 (0,15–1,53)	0,944 (0,67–1,34)	1,127 (0,99–1,28)
Inactivos	4,034 (1,42–11,49)^c^	2,191 (1,26–3,80)^c^	2,290 (1,99–2,63)^c^	1,772 (1,67–1,88)^c^
Ocupados	Ref.	Ref.	Ref.	Ref.

Nota: F (Archer y Lemeshow): Inmigrantes: modelos SAV = 0,764; modelo tratamiento = 0,000; Locales: modelos SAV = 0,000; modelo tratamiento = 0,000. Los modelos ajustados por variables sociodemográficas se estimaron sin considerar la constante.

Etnia: considera solo a los 9 pueblos originarios reconocidos por ley en Chile; Ocupación: ocupado (tener trabajo), desocupados (no tener trabajo, pero no se interesa en tenerlo) e inactivos (no tiene interés en trabajar).

Modelo SAV: salud autoevaluada ~ discriminación + migración + participación social + redes de apoyo social + variables sociodemográficas. Modelo Tratamiento: Estar o haber estado en tratamiento médico ~ discriminación + migración + participación social + redes de apoyo social + variables sociodemográficas.

OR: ; IC95%: intervalo de confianza del 95%; SAV: salud autoevaluada; Ref.: referencia.

^a^ p-valor OR < 0,05 prueba de Wald, modelo ajustado solo por la variable discriminación.

^b^ p-valor OR < 0,05 prueba de Wald, modelo ajustado solo por las variables discriminación, participación social y redes de apoyo.

^c^ p-valor OR < 0,05 prueba de Wald, modelo ajustado solo por las variables discriminación, participación social, redes de apoyo y variables sociodemográficas.

Respecto al acceso a atención en salud, la interacción entre discriminación y migración no fue significativa en la falta de consulta ante enfermedad o accidente (p-valor OR _>_ 0,05). Sin embargo, en población consultante, inmigrantes (OR = 0,454; IC95% 0,21–0,99) y chilenos (OR = 0,477; IC95% 0,27–0,86) no discriminados tuvieron significativamente menos chance de presentar problemas durante la consulta comparados con inmigrantes discriminados. Ya sea que perciban discriminación o no, los inmigrantes presentaron más chance de no tener previsión de salud en comparación con los nacidos en Chile. Específicamente, los chilenos discriminados tienen 0,265 (IC95% 0,17–0,41) veces menos chance de no tener previsión de salud en comparación con inmigrantes discriminados, en chilenos no discriminados este valor fue de 0,212 (IC95% 0,14–0,32) veces (Figura 1C).

Respecto a los factores sociodemográficos en relación al acceso a atención en salud en inmigrantes, la pertenencia étnica resultó ser significativa en la falta de previsión de salud (OR = 0,565) y tenencia de seguros complementarios (OR = 5,801). Asimismo, la zona de residencia fue significativa para la falta de previsión de salud (OR = 1,545), tenencia de seguros complementarios (OR = 2,181), falta de consulta (OR = 0,181) y problemas durante el proceso de consulta (OR = 0,233). Finalmente, el pertenecer el quintal más rico en comparación al quintil más pobre resultó significativo en la falta de seguros complementarios (OR = 0,129) y la no consulta (OR = 0,124) (Tabla 4).

**Tabla 4:** Modelos de regresión logística, Tener sistema previsional de salud (sí/no), algún miembro de la familia cubierto por seguros de salud complementario (sí/no), no consulta ante enfermedad o accidente (sí/no), problemas durante la consulta (sí/no), ajustado por discriminación auto-informada, capital social y características sociodemográficas, en inmigrantes y locales. Chile, 2017.

			Inmigrantes			Locales		
		
	Modelo SPS	Modelo SSC	Modelo CS	Modelo CCP	Modelo SPS	Modelo SSC	Modelo CS	Modelo CCP
							
	OR (IC95%)	OR (IC95%)	OR (IC95%)	OR (IC95%)	OR (IC95%)	OR (IC95%)	OR (IC95%)	OR (IC95%)
Discriminación								
Sí	0,569 (0,30–1,09)	0,728 (0,46–1,15)	0,596 (0,24–1,46)	1,399 (0,60–3,28)	1,123 (0,90– 1,40)^a,b^	0,940 (0,84–1,05)^a,b^	1,098 (0,87–1,39)	1,607 (1,41–1,83)^a,b,c^
Participación social								
Sí	0,713 (0,42–1,20)	0,970 (0,63–1,49)^b^	1,535 (0,62–3,79)	1,601 (0,82–3,14)	0,798 (0,68–0,94)^b,c^	0,914 (0,85–0,98)	0,914 (0,76–1,09)	1,089 (0,98–1,20)^b^
Redes de apoyo								
Sí	0,833 (0,47–1,49)^b^	2,345 (1,37–4,03)^b,c^	2,528 (0,98–6,50)^b^	0,212 (0,07–0,69)^b,c^	0,221 (0,18–0,27)^c^	2,264 (1,99–2,58)^b^	0,284 (0,21–0,38)^b,c^	0,481 (0,39–0,59)^c^
Sexo								
Mujer	1,050 (0,46–2,42)	1,345 (0,87–2,07)	1,152 (0,50–2,66)	1,888 (0,87–4,08)	0,543 (0,46–0,63)^c^	1,329 (1,23–1,43)	0,742 (0,63–0,88)^c^	1,082 (0,98–1,20)
Edad								
	0,988 (0,97–1,01)	0,993 (0,98–1,01)	0,995 (0,97–1,02)	1,007 (0,99–1,03)	0,977 (0,97–0,98)^c^	1,017 (1,01–1,02)	0,991 (0,99–1,00)^c^	0,999 (1,00–1,00)
Etnia								
Sí	0,565 (0,33–0,97)^c^	5,801 (3,53–9,53)^c^	0,386 (0,514–1,03)	0,576 (0,13–2,51)	0,641 (0,55–0,75)^c^	1,020 (0,91–1,14)	0,741 (0,60–0,91)^c^	0,704 (0,60–0,82)^c^
Nivel educacional								
Sin educación.	0,473 (0,10–2,15)	-	-	-	0,477 (0,26–0,88)^c^	8,022 (5,30–12,14)	0,576 (0,34–0,98)^c^	1,078 (0,79–1,46)
Básica	0,891 (0,40–2,01)	3,881 (1,49–10,08)^c^	0,240 (0,05–1,11)	0,899 (0,30–2,68)	0,699 (0,55–0,89)^c^	4,494 (3,98–5,08)	0,883 (0,67–1,17)	1,214 (1,04–1,42)^c^
Media	0,641 (0,34–1,21)	2,805 (1,51–5,20)^c^	0,363 (0,15–0,85)^c^	1,299 (0,58–2,91)	0,752 (0,61–0,93)^c^	2,357 (2,18–2,55)	0,895 (0,71–1,13)	1,124 (0,99–1,28)
Zona								
Rural	1,545 (1,06 – 2,25)^c^	2,181 (0,95 – 5,01)^c^	0,181 (0,05 – 0,67)^c^	0,233 (0,12 – 0,46)^c^	1,043 (0,86–1,26)	1,350 (1,16–1,57)	1,138 (0,93–1,39)	0,879 (0,74–1,04)
Quintil de ingreso								
II	0,595 (0,33–1,08)	1,794 (0,79–4,07)	0,154 (0,03–0,84)^c^	1,149 (0,31–4,21)	0,737 (0,60–0,90)^c^	0,749 (0,66–0,84)	0,804 (0,65–0,99)^c^	0,898 (0,78–1,03)
III	0,693 (0,35–1,36)	1,197 (0,61–2,37)	0,804 (0,21–3,03)	0,766 (0,25–2,39)	0,768 (0,63–0,94)^c^	0,548 (0,49–0,62)	0,802 (0,65–1,00)^c^	0,822 (0,70–0,96)^c^
IV	1,087 (0,48–2,47)	0,570 (0,29–1,12)	0,425 (0,10–1,78)	0,609 (0,16–2,38)	0,690 (0,54–0,87)^c^	0,367 (0,33–0,41)	0,733 (0,55–0,98)^c^	0,736 (0,63–0,86)^c^
V (más rico)	0,514 (0,26–1,03)	0,129 (0,08–0,22)^c^	0,124 (0,02–0,65)^c^	0,771 (0,19–3,19)	0,701 (0,51–0,96)^c^	0,173 (0,15–0,19)	0,577 (0,41–0,80)^c^	0,528 (0,43–0,64)^c^
Ocupación								
Desocupado	1,939 (0,98–3,84)	1,530 (0,47–4,94)	-	3,322 (1,16–9,48)^c^	1,593 (1,17–2,16)^c^	2,180 (1,71–2,78)	0,689 (0,40–1,19)	0,987 (0,75–1,30)
Inactivo	1,070 (0,50–2,28)	0,614 (0,31–1,22)	0,435 (0,13–1,46)	0,437 (0,13–1,52)	0,666 (0,53–0,84)^c^	1,375 (1,23–1,54)	0,971 (0,77–1,22)	1,146 (1,00–1,31)^c^

Nota: Modelo SPS: Tener sistema previsional de salud ~ discriminación + migración + participación social + redes de apoyo social + variables sociodemográficas. SSC: Algún miembro de la familia cubierto por seguros de salud complementario ~ discriminación + migración + participación social + redes de apoyo social + variables sociodemográficas. Modelo CS: No consulta ante enfermedad o accidente ~ discriminación + migración + participación social + redes de apoyo social + variables sociodemográficas. Modelo CCP: Problemas durante la consulta ~ discriminación + migración + participación social + redes de apoyo social + variables sociodemográficas.

Categorías de referencia: discriminación, no discriminado; participación social, no participa; redes de apoyo, sin redes; sexo, hombre; etnia, no pertenece a ninguna; nivel educacional, superior; zona, urbana; quintil, I (más pobre); ocupación, ocupados.

OR: *odds ratio*; IC95%: intervalo de confianza del 95%; SPS: sin sistema previsional de salud; SSC: sin miembros del hogar con seguro complementario de salud; CS: no consulta ante enfermedad o accidente; CCP: consulta con problemas (solo en población consultante).

^a^ p-valor OR < 0,05; prueba de Wald, modelo discriminación.

^b^ p-valor OR < 0,05; prueba de Wald, modelo discriminación y capital social.

^c^ p-valor OR < 0,05 ; prueba de Wald, modelo ajustado solo por las variables discriminación, participación social, redes de apoyo y variables sociodemográficas.

En contraste con los inmigrantes, en locales la discriminación fue significativa en el presentar problemas durante el proceso de consulta, en desfavor de los discriminados [OR_inmigrantes_ = 1,399 (IC95% 0,60–3,28) *versus* OR_locales_ = 1,604 (IC95% 1,41–1,83)] (Tabla 4).

Finalmente, en inmigrantes un mayor tiempo de residencia en el país fue un factor de riesgo significativo en el estar o haber estado en tratamiento médico (OR = 1,067) y protector en el no tener previsión de salud, SSC (OR = 0,922) y falta de seguros de salud complementarios (OR = 0,974). En este último el país de origen también resultó ser un factor significativo – (resultado no presentado en tabla).

## DISCUSIÓN

Este estudio buscó describir brechas de desigualdad en la percepción de discriminación entre inmigrantes y locales, desde un enfoque de inequidad social en salud. Los resultados presentados sugieren que inmigrantes residentes en Chile tienen mayor chance de percibir discriminación comparado con locales, y que la interacción entre inmigración y discriminación se puede relacionar con peores resultados de salud autoevaluada y tratamiento médico. Acorde a la evidencia, la discriminación percibida podría desencadenar una serie de mecanismos que intervienen en el riesgo de enfermar, pudiendo distinguirse entre (i) directos, provocando una respuesta al estrés y afrontamiento poco saludable de este; e (ii) indirectos o estructurales, al limitar el empleo, ingreso, bienestar general, y acceso y trato percibido en sistemas y servicios de salud, entre otros^12^. Respecto a este último, si bien en este estudio la discriminación no mostró una asociación significativa con la no consulta, en algunas situaciones sí se relacionó con el presentar problemas durante la consulta, pudiendo tener repercusiones negativas en la búsqueda de atención en el futuro.

Algunos estudios internacionales han mostrados resultados similares. Por ejemplo, en refugiados e inmigrantes iraquíes la percepción de discriminación contribuyó significativamente a la predicción de depresión y mala o regular salud autoevaluada, aumentando hasta dos veces el riesgo en población discriminada^17^. En inmigrantes ghaneses en Europa niveles más altos de discriminación percibida se asociaron con un alto riesgo de enfermedades cardiovasculares^24^, y en inmigrantes usuarios de diverso origen de servicios sociosanitarios en Barcelona aproximadamente el 50% de los encuestados indicó sentirse discriminado en tales servicios, empeorando en inmigrantes irregulares^25^.

En el contexto actual, la discriminación tiende a ocurrir como microagresiones sutiles o estructurales, en vez de explicita y abierta. Así, la discriminación percibida ha sido utilizada mundialmente en estudios similares, ya que su naturaleza subjetiva permite capturar eventos no definidos como discriminatorios según leyes locales o definiciones sociales particulares^11,12^. En este sentido, en población inmigrante, el capital social puede jugar un rol ambiguo en la percepción de discriminación al proteger frente a eventos discriminatorios y su afrontamiento, pero puede facilitar la percepción de agresiones sutiles acorde a las costumbres del país receptor y el aislamiento en grupos cerrados.

En Chile, las experiencias de discriminación se entrelazan con otras formas de rechazo, exclusión social, clasismo, racismo y desprecio a personas en pobreza^21^. Entre los participantes del estudio que percibieron discriminación aproximadamente un tercio afirmó ser discriminados por múltiples motivos. Esto da cuenta de la complejidad de estos procesos sociales, múltiples y dinámicos, así como la importancia de contar de manera coordinada y planificada con acciones de protección de la dignidad, salud y cualquier forma de desventaja social en inmigrantes. Considerando los posibles efectos crónicos de la discriminación y que los principales motivos de discriminación en inmigrantes se perciben en apariencia externa, es urgente concientizar a la población local acerca de su prevención y rechazo, sobre todo en la atención en salud.

Las fortalezas de este estudio consisten en el análisis simultáneo de variables sociales y de salud, y la utilización de una base poblacional confiable y altamente aceptada en Chile, permitiendo aceptable precisión general. Por otra parte, al tratarse de un análisis de datos secundarios, son limitaciones la exclusión de grupos de interés (residentes de áreas inaccesibles y personas en situación de calle), la imposibilidad de analizar otras variables pertinentes a la problemática y tamaños de muestra limitados en población migrante al no ser foco de selección de la muestra. Finalmente, se reconoce la naturaleza transversal de los datos impidiendo inferir causalidad.

Los resultados de este estudio pueden apoyar en el desarrollo de políticas públicas y de salud en Chile y en otros países que muestren resultados similares. Existe evidencia en América Latina y el Caribe que documenta la relación entre experiencias de estigma, discriminación y racismo y peores resultados de salud en migrantes, refugiados y personas en movimiento. Estos fenómenos sociales son complejos y dinámicos, y relevantes a la hora de profundizar en los mecanismos sociales de rechazo y exclusión que desencadenan una serie de resultados de salud subjetivos y objetivos. La discriminación tiene su origen en la formación de grupos sociales que se autoconciben de manera distinta a los demás, creando así una o múltiples “otredades” de menor rango o casta, las cuales están cargadas de estereotipos y prejuicios. En este sentido, la mayor percepción de discriminación en migrantes comparados a locales en Chile ilustra la existencia de patrones de interacción social que va diferenciando, y subyugando, al migrante por su estatus de extranjero, además, como sugieren los resultados de este estudio, de su color de piel y aspecto físico. En materia de política pública, este estudio permite reforzar la importancia de atender, contener y revertir procesos de estigma y discriminación hacia migrantes en Chile, que también se ha documentado en otros países. Dichas recomendaciones de política pública pueden organizarse en torno a niveles diferenciados de acción, por ejemplo: (i) nivel político, con el desarrollo de normativas y mecanismos de rechazo a toda forma de discriminación en contextos públicos, incluyendo en sistema de salud, (ii) nivel comunitario, mediante campañas de sensibilización que promuevan el respeto a la diversidad social y cultural y la construcción de comunidades inclusivas y respetuosas, (iii) sistema de salud, mediante desarrollo de mecanismos de formación continua en la temática de migración, discriminación y salud, la promoción de equipos con competencias interculturales en salud y campañas permanentes de rechazo explícito y auditoría a toda forma de discriminación y violencia en la atención de salud.

Sobre futuras investigaciones, existen tres aspectos relevantes documentados en la literatura que no están suficientemente analizados en este estudio: (i) variables migratorias críticas para la experiencia de discriminación como regularidad y refugio/asilo^13^, (ii) enfoque longitudinal de curso de vida y (iii) análisis de problemas específicos de salud. Asimismo, un enfoque de desigualdades sociales que integre la intersección con identidad de género es relevante, pues estas suelen derivarse de valores dominantes de la sociedad de acogida y se exacerban en mujeres^4^. Por tanto, este estudio abarca múltiples enfoques, pero puede seguir profundizándose, especialmente en encuestas poblacionales que incluyan una adecuada representación de colectivos migrantes.
